# Hepatitis C—Everything a Primary Care Physician Needs to Know About Diagnosis, Management, and Follow-Up †

**DOI:** 10.3390/jcm14134801

**Published:** 2025-07-07

**Authors:** Sindhuri Benjaram, Shweta Kapur, Anusha McKay, Mohamad Khaled Almujarkesh, Kassandra S. Carter, Alexandra Picardal, Diane Levine, Prateek Lohia

**Affiliations:** 1 Thomas Memorial Hospital, West Virginia University, Charleston, WV 26506, USA; 2 School of Health Sciences, Oakland University, Rochester, MI 48309, USA; 3 Department of Internal Medicine, Wayne State University School of Medicine, Detroit, MI 48201, USA; 4 Department of Gastroenterology, Advent Health, Orlando, FL 32803, USA; 5 Department of Internal Medicine, TriStar Centennial Medical Center, Nashville, TN 37203, USA; 6 Department of Emergency Medicine, Henry Ford Hospital, Detroit, MI 48202, USA

**Keywords:** Hepatitis C, direct-acting antivirals, primary care physician, Hepatitis C screening, cost of treatment

## Abstract

Hepatitis C virus (HCV) infection is a major public health concern, with more than 58 million people chronically infected worldwide. The management of HCV, once the domain of specialists only, has been revolutionized by the advent of direct-acting antiviral therapies. To reduce the burden of HCV in the United States (US), emphasis is now being placed on the involvement of primary care physicians in the management of HCV patients. Inclusion of more primary care providers in the HCV diagnosis and treatment initiatives can assist in achieving the goal of HCV elimination, especially in the medically underserved areas. To actively engage in the management of HCV, primary care providers must understand its epidemiology, risk factors, natural history, current treatment regimen, and potential complications. This manuscript reviews these key areas, along with presenting the cost-effectiveness of treatment and evidence-based guidelines for follow-up care in adults with chronic HCV infection who have undergone HCV treatment. Equipped with this foundational knowledge about HCV management, primary care physicians can play a vital role in eliminating HCV.

## 1. Introduction

First identified in 1988, the hepatitis C virus (HCV) is a single-stranded RNA virus that has since emerged as a significant global health concern. At least six major HCV genotypes have been identified, with genotype 1 accounting for about 46% of infections worldwide [1]. Genotypes 1–3 are prevalent worldwide, whereas genotypes 4 and 5 are found mostly in Africa, and genotype 6 is prevalent in Asia [2]. Globally, an estimated 58 million people are living with chronic HCV, including approximately 2 to 2.2 million individuals in the United States (US) [3,4]. In 2016, the World Health Organization (WHO) adopted a strategy to eliminate viral hepatitis C as a public health threat by 2030 [5]. This was defined as a 90% reduction in new chronic infections and a 65% reduction in mortality as compared to 2015 [6]. The elimination of HCV as a public health threat would require 90% of all the individuals with HCV to be diagnosed and at least 80% of the diagnosed individuals to be treated [6].

Acute HCV infection is either asymptomatic or presents with mild, nonspecific symptoms. Without treatment, 15–45% of individuals spontaneously clear acute infections within 6 months of onset, and the remaining individuals develop chronic HCV [6,7]. Chronic hepatitis C is associated with significant morbidity, mortality, and economic burden [8]. It can progressively lead to cirrhosis, liver failure, and hepatocellular carcinoma (HCC).

The hepatitis C virus (HCV) genome is a ~9.6 kb single-stranded RNA that encodes structural proteins (Core, E1, E2), the p7 ion channel, and nonstructural proteins (NS2, NS3, NS4A, NS4B, NS5A, and NS5B) [9,10]. These proteins are essential for viral entry, replication, and assembly, and thus they serve as key targets for DAAs [11]. Current DAA regimens are categorized into three major classes: NS3/4A protease inhibitors, NS5A inhibitors, and NS5B RNA-dependent RNA polymerase inhibitors (e.g., nucleotide analogs) [12]. Given the high genetic variability of HCV and the presence of multiple genotypes and subtypes, combination therapy using agents from different classes is recommended to prevent resistance and achieve a sustained virologic response (SVR) [13]. This approach parallels the rationale behind combination antiretroviral therapy in HIV management. Moreover, suboptimal dosing or poor adherence can promote the emergence of drug-resistant variants, highlighting the importance of proper regimen selection and patient monitoring during treatment.

## 2. Role of Primary Care Physicians

Traditionally HCV patients were primarily managed by gastroenterologists and infectious disease (ID) specialists. Early diagnosis of HCV infection, linking HCV patients to proper care, and adherence to treatment are pivotal for HCV elimination [14]. Historically, very few primary care physicians (PCPs) have been involved in the management of HCV patients, especially in rural areas, likely due to lack of training [15]. However, the transformation of HCV treatment has occurred with the advent of DAA drugs. This, coupled with the limited availability of specialists treating HCV, has resulted in increased calls for the involvement of primary care providers in chronic HCV treatment and follow-up [16,17,18]. Studies have reported similar rates of achieving a sustained virological response with DAA treatment, irrespective of whether the treatment was administered by ID specialists or trained primary care providers [19]. Multiple studies have also shown that with education and training, PCPs can safely assume HCV treatment meeting the need for HCV care in rural areas with limited availability of specialist care [20,21,22]. Active engagement of primary care physicians in the management of patients with chronic HCV is crucial in tackling the HCV epidemic.

## 3. Objective

This manuscript aims to provide readers with foundational knowledge about HCV diagnosis, management, and follow-up. This information can prepare primary care physicians to play an active role in the early diagnosis and management of adult patients with chronic HCV. The material presented in this manuscript can also serve as an initial point of reference for primary care physicians interested in independently treating chronic HCV patients. This paper was also presented as a poster at the annual Society of General Internal Medicine conference in Boston, 2024 [23]. 

## 4. Prevalence of HCV Worldwide

The annual incidence of acute HCV has been estimated to be around one million globally [24]. The prevalence of HCV decreased from 2.8% (185 million) to 2.5% (177 million) from 1990 to 2005, with the most notable decreases in the high-income countries [25]. The highest prevalence (>3.5%) was found to be in Central Asia and Central Africa, and the lowest prevalence (<1.5%) in South Africa, North America, Central Latin America, Pacific Asia, and Western and Central Europe [25].

## 5. Prevalence and Mortality of HCV in the United States (US)

In the US, an estimated 2 to 2.2 million people are chronically infected with HCV [3,4]. HCV prevalence is higher in males (1.4%) compared to females (0.5%), with non-Hispanic Whites having the highest prevalence [4]. In 2020, the mortality rate due to HCV was 3.45 per 100,000 US population, showing a decline from its peak of 5.03 per 100,000 in 2013 [26]. This downward trend in HCV mortality rates since 2013 is driven by substantial decreases in HCV deaths in the West, Southwest, and Northeast regions of the US [27]. One potential factor resulting in this decrease is the emergence of curative treatment in the form of DAA drugs in 2011 [27].

## 6. HCV Routes of Transmission

Hepatitis C can be transmitted through contact with infected blood or via sexual intercourse [1]. In 15–45% of people exposed to hepatitis C virus, the infection is self-limited [6,7]. With self-limited infection, HCV-specific antibodies, not RNA, can be detected in blood. However, in the remaining population, HCV infection persists with long-term complications, including cirrhosis that can eventually develop into HCC [28]. HCV patients may often present with hepatitis B virus (HBV) or human immunodeficiency virus (HIV) coinfection due to similar transmission routes [29].

Intravenous (IV) drug use is the strongest risk factor for transmission of infection, with other significant risk factors including multiple sexual partners, tattoos, hemodialysis, and perinatal transmission [1]. Although sexual transmission accounts for a minority of HCV cases, acute hepatitis C or reinfection has become a significant problem in HIV-infected men who have sex with men [2].

## 7. Diagnosis and Screening for HCV

Anti-HCV antibody testing is recommended under all guidelines as the first step to screen for chronic HCV infection [30]. Seroconversion with detectable anti-HCV antibodies normally occurs 32 to 46 days after initial infection [31], although it may take as long as 12 to 24 weeks particularly in immunocompromised individuals [32].

In patients with acute infection or an impaired immune response, HCV RNA testing is recommended over anti-HCV antibody testing. This decreases the possibility of false negatives [33,34]. HCV RNA is detectable within 1–2 weeks of acute infection [35].

**Figure 1:**
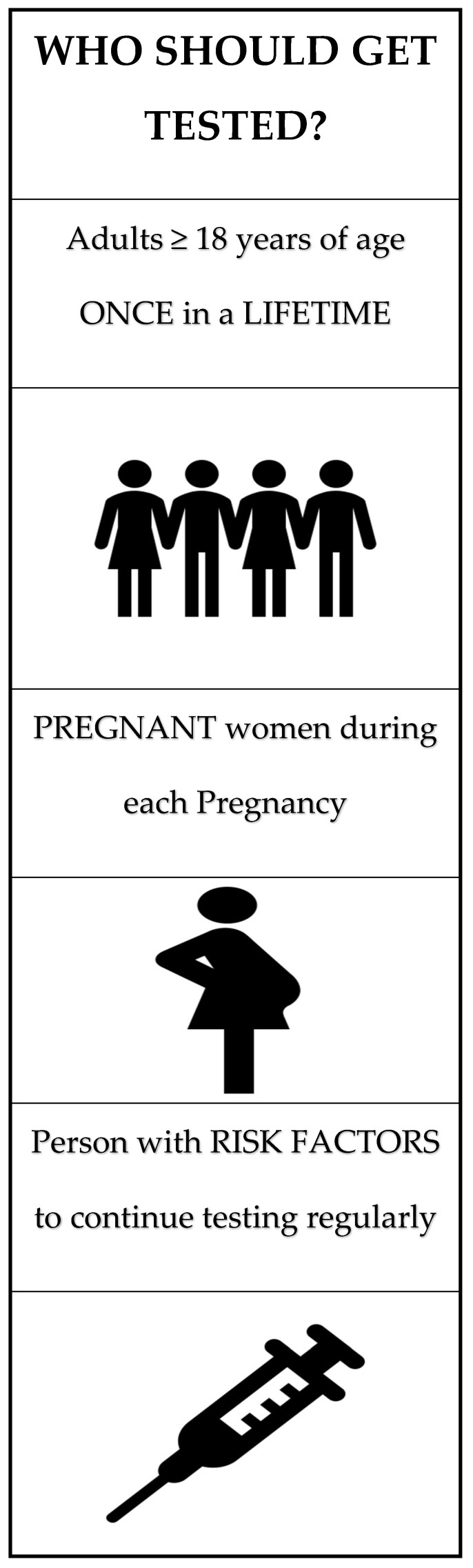
Hepatitis C screening criteria as per the CDC and USPTF guidelines. Figure created by authors using stock images from Microsoft Word.

**Figure 2:**
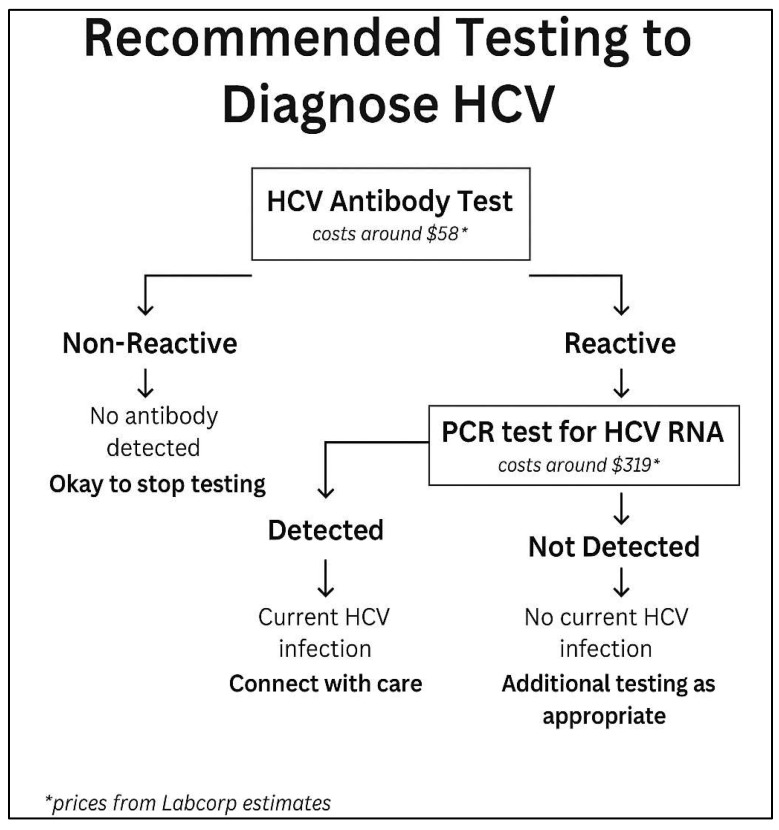
Flowchart depicting steps to diagnose hepatitis C infection and if patient needs to be referred for further treatment. Prices from Labcorp estimates are based on walk-in testing. Available online: https://www.findlabtest.com/lab-test/search?q=lt140659+lt550080 (10 December 2023).

Universal testing is now recommended by the U.S. Preventive Services Task Force (USPSTF), the American Association for the Study of Liver Diseases (AASLD), the European Association for the Study of the Liver (EASL), and the Centers for Disease Control and Prevention (CDC). Further details outlining the screening criteria and testing to diagnose HCV are shown in Figure 1 and Figure 2, respectively.

## 8. Natural History of Infection

**Figure 3:**
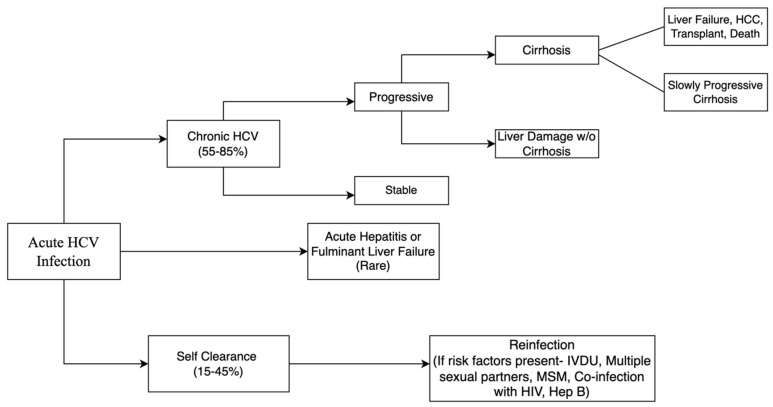
Flowchart depicting natural history of acute hepatitis C infection and its progression.

The course of natural history of HCV infection is shown in Figure 3. Acute HCV is clinically mild, often goes unrecognized and undiagnosed, and is spontaneously cleared in 15–45% of patients [6,7]. Additional follow-up for reinfections might be needed depending on what risk factors a patient may have [6,7]. Although acute HCV infection is frequently asymptomatic, it can manifest as acute hepatitis with jaundice, or in rare cases, as fulminant hepatic failure [36]. Alanine transaminase (ALT) levels frequently reach values more than two times the normal upper limit with concomitant rises of serum bilirubin [31]. Thus, patients can develop clinical symptoms two to twelve weeks after transmission [24]. Even in symptomatic patients, symptoms and signs are nonspecific, and commonly reported symptoms include fatigue, nausea, abdominal pain, loss of appetite, mild fever, itching, or myalgias [24]. Development of jaundice occurs in 50–84% of clinically symptomatic patients with acute HCV infection [37].

## 9. Complications of HCV Infection

### 9.1. Hepatic

The most serious complication of chronic HCV infection is liver-related mortality due to decompensated liver cirrhosis or the development of HCC [32]. It has been previously reported that 16–33% of the patients developed liver cirrhosis within 20 years of initial infection [32,38]. There are certain risk factors associated with an increased risk of progression to cirrhosis and subsequently HCC. Advanced age is one of the most important risk factors for fibrosis progression, and other factors include increased alcohol consumption, coinfection with HBV or HIV, and male sex [32,39]. Assessing the degree of fibrosis is recognized as an important predictor of HCV disease progression and clinical outcomes. Patients with bridging fibrosis are at an increased risk of developing complications from advanced liver disease such as portal hypertension [40]. On the other hand, HCV infection acquired during childhood seems to take a much milder course [41]. The mechanism to explain this has not been fully elucidated; however, changes in the regenerative capacity of the liver, alterations of the immune system, and telomere shortening with age may play important roles in this mechanism [42].

### 9.2. Extrahepatic

Patients who develop chronic HCV infections have a high prevalence of multiple extrahepatic conditions compared to age-matched, general-population controls [43]. HCV infection is strongly associated with mixed cryoglobulinemia vasculitis [44]. There are also complex associations between HCV and other conditions such as diabetes, thyroid disease, chronic renal disease, lichen planus, porphyria cutanea tarda, and neuropsychiatric conditions such as depression [45].

## 10. History of HCV Treatment

Over the last 20 years, antiviral therapy for HCV infection has drastically improved. Since 2013, the availability of DAAs has increased both the response rates and tolerability to HCV treatment [46]. They can be orally administered and treat a larger spectrum of viral genotypes with shorter treatment durations [47]. In addition, they can be prescribed without intramuscularly administered interferon (IFN), which has been associated with adverse side effects. The cure rate with the DAAs is higher than 95% [48,49]. The remarkable clinical efficacy of DAAs has resulted in their adoption as a public health intervention to control HCV infection globally.

## 11. Considerations for Primary Care Physicians Before Initiating HCV Treatment

There are several considerations to account for prior to initiating HCV treatment to ensure its safety and effectiveness. The first is determining whether the patient has active or chronic HBV infection. During or after DAA therapy in HCV patients, many cases of HBV reactivation have been reported in patients with HBV who were not receiving HBV suppressive therapy [50]. Thus, hepatitis B surface antigen (HBsAg), total antibody to hepatitis B core antigen (anti-HBc), and hepatitis B surface antibody (anti-HBs) testing should be obtained in all individuals before initiating treatment [50]. Subsequent immunization with HBV vaccines should be considered for those eligible. Of note, being HBsAg-positive is not a contraindication to DAA therapy. Those who test positive for HBsAg should be further tested for HBV DNA. For patients with a positive HBV DNA test and/or evidence of active HBV infection, HBV therapy should be started concurrently or before the initiation of DAA therapy [51]. A negative HBsAg test with positive hepatitis B core antibodies and +/− hepatitis B surface antibodies indicate mostly resolved HBV infection, and the risk of reactivation of HBV with DAAs is relatively low [52].

Secondly, drug interactions should also be considered. Medications to be aware of include proton pump inhibitors, statins, amiodarone, and St. John’s wort extract [53]. Generally, current therapies have well-characterized pharmacology and manageable drug interaction profiles. Of note, no modifications are needed for patients receiving medication-assisted therapy (MAT) for opioid use disorder [54]. However, clinicians should work closely with addiction specialists to manage any potential medication interactions and ensure that patients have adequate support during treatment [54,55]. The common side effects of DAA therapy include fatigue, nausea, diarrhea, and vomiting. Although rare, serious side effects including pneumonia, renal failure, myocardial infarction, and cardiac arrest have been reported [56].

## 12. Current HCV Treatment Guidelines

The goal of hepatitis C treatment is to attain SVR. SVR refers to the continued absence of HCV from the blood, thus reflecting the eradication of HCV from the body. SVR after 12 weeks of treatment is a commonly used parameter to gauge the success of HCV treatment [57,58]. In 2011, the first DAA agents, boceprevir and telaprevir, were approved and primarily used for treatment of genotype 1. However, in 2013, simeprevir and sofosbuvir became the first once-daily DAAs approved for HCV treatment. These newer DAAs were determined to have pan-genomic properties with more efficacy, a shorter course, better tolerance, and higher rates of SVR [49]. Compared to the historical treatment with pegylated interferon (PEG-IFN) + ribavirin, which resulted in 40–50% of patients achieving a sustained virologic response (SVR), DAAs have been shown to produce an SVR in over 95% of patients [48,49].

The benefits of HCV treatment include a decreased likelihood of the development or progression of HCV complications (cirrhosis, liver failure, HCC) [59], improvement in extrahepatic complications [60], and a reduction in the transmission of HCV, especially in high-risk populations, which could then decrease the disease burden in these populations [49]. It should be noted that specific contraindications and warnings for each patient should be considered when choosing a treatment regimen. Many studies suggest not initiating HCV treatment in patients with less than 12 months of life expectancy [61,62]. The different treatment regimens based on HCV genotype have been summarized in Table 1.

## 13. Cost of Hepatitis C and Its Treatment

Due to the prevalence of hepatitis C, the estimated cost of HCV treatment in 2011 was USD 6.5 billion with a range of USD 4.2 to USD 8.2 billion. The total cost was expected to peak in 2024 at USD 9.1 billion with a range of USD 6.4 to USD 13.3 billion [63]. This peak was attributed to more advanced liver disease, with decompensated liver cirrhosis accounting for half of the peak [63].

**Figure 4:**
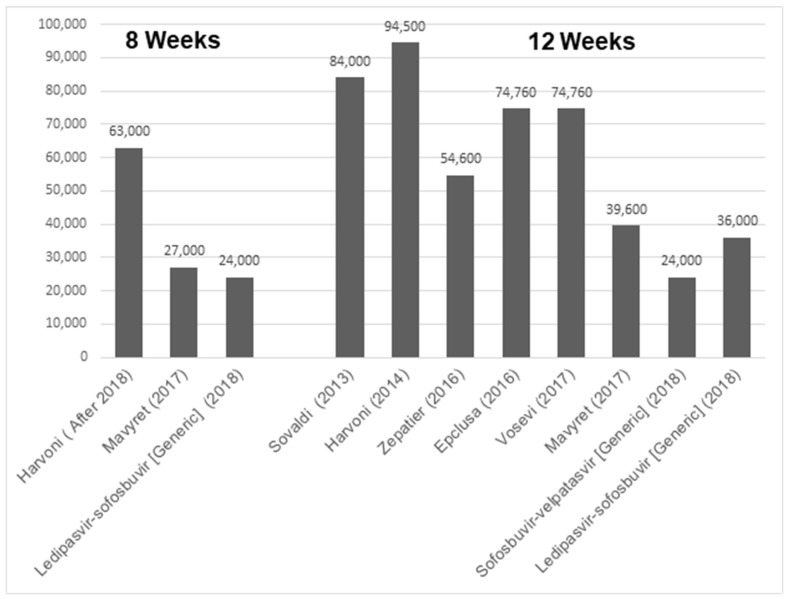
Bar graph indicating gross cost of different HCV drugs based upon either 8 or 12 weeks of treatment regimen [64].

Although DAAs were expensive to begin with, their cost has been constantly decreasing over the years, due to the availability of generic versions and competition. For example, when Sovaldi (sofosbuvir) was the first DAA introduced in 2013, the cost of a 12-week treatment course for HCV was USD 84,000 [64]. However, the current prices for Mavyret (glecaprevir/pibrentasvir) and sofosbuvir/velpatasvir (generic) are listed at USD 39,600 and USD 24,000, respectively (Figure 4) [65]. Thus, compared to the cost of the burden of disease, the treatment of HCV proves cost-effective [66].

Chronic HCV-associated liver cirrhosis is one of the leading causes of liver transplant, accounting for around 50% of the liver transplant cases in US [67,68]. A systematic review found that liver transplantation in the US totals about USD 163,438 in the US, compared to USD 103,548 for other developed nations, and the subsequent yearly care is also costly [69]. Thus, HCV screening and early treatment are a highly cost-effective strategy for reducing the HCV burden in the United States [70].

## 14. Summary of Steps PCPs Should Take in HCV Detection and Treatment

Primary care physicians (PCPs) are essential to the detection, diagnosis, and treatment of HCV. Those without access to care are 19 times more likely to be unaware of their HCV infection [71]. All patients should receive HCV screening at least once in their lifetime, and high-risk patients like those who inject drugs should receive periodic testing [72]. The first step to screening is anti-HCV antibody testing [30]. Because anti-HCV antibodies can remain high in limited acute infection, patients who test positive should be tested for HCV RNA [30]. Commonly used assays include Roche COBAS TaqMan Version 1.0, Roche COBAS TaqMan Version 2.0, and the Abbott RealTime HCV (ART) assay [73]. After a positive test for HCV RNA, comprehensive baseline laboratory values should be obtained. These include a CBC, hepatic function panel (albumin, total and direct bilirubin, alanine transaminase, aspartate aminotransferase, and alkaline phosphatase), International Normalized Ratio (INR), kidney function tests (Cr, GFR), baseline HCV RNA levels, HCV genotype, assessment for coinfection with HIV or HBV, and a serum pregnancy test in women of childbearing age [72]. Noninvasive biomarkers like APRI (Aspartate Transaminase to Platelet Ratio Index), Fibrosis-4 (FIB 4) Index for Liver Fibrosis, Fibrospect II, and HCV Fibrosure and imaging studies like Transient Elastography by Fibroscan are used for screening and staging fibrosis. Assessment of fibrosis helps determine the prognosis of the disease, select patients for treatment, and monitor the success of treatment [74]. Before initiation of treatment, a complete medical history must be obtained from the patient, evaluating risk factors for chronic liver disease, other chronic diseases like diabetes mellitus, family history of any cancer or liver disease, use of any illicit drugs, history of alcohol consumption, and a complete medication list including any herbal supplements [75]. Coadministration of amiodarone and sofosbuvir-containing regimens is not recommended due to potential severe bradyarrhythmic effects [76]. At 12 weeks post-treatment, long-term HCV cure is said to be achieved if HCV RNA levels are undetectable, known as SVR 12 [77]. As per the recent AASLD and IDSA guidelines, routine HCV testing during DAA treatment is not recommended unless a patient’s ALT levels do not decline (when elevated before initiation of therapy) or adherence to DAA is questionable [78]. In patients who have achieved SVR, virologic relapse is rare beyond 12 weeks after completion of therapy [79,80]. However, repeat HCV RNA testing can be considered 24 weeks or more after treatment in patients with ALT increased above the normal limits [78].

## 15. Follow-Up for Patients with Cirrhosis

After a patient has achieved SVR 12 post-treatment, follow-up is recommended for patients with concomitant cirrhosis [81]. In patients with cirrhosis, even after achieving SVR 12, an abdominal ultrasound with or without alpha fetoprotein (AFP) measurement every 6 months and upper endoscopy every 2–3 years is recommended for life [82]. PCPs should continue standard lifestyle risk factor screening and motivational interviewing when warranted. Discussion regarding the avoidance of alcohol, tobacco, and injected drug use should take place to prevent additional hepatic or extrahepatic manifestations [72]. HCV RNA testing is recommended in patients who have an increase in ALT or AST at any time and in those who continue to partake in behaviors that would make them susceptible to liver damage [2]. Failure to follow up with patients and confirm SVR 12 can lead to serious consequences. The risk is even higher in those with existing liver fibrosis, and due to the high risk of HCC, these patients need regular monitoring even after being treated for HCV. Thus, it is crucial for PCPs to provide adequate follow-up care and provide education to patients to prevent adverse outcomes in this population.

## 16. Follow-Up for Patients Without Cirrhosis

In patients who do not have a diagnosis of cirrhosis, there is no recommended follow-up [82]. However, patients with high-risk behaviors should be periodically screened for HCV RNA to detect reinfection [82].

## 17. Treatment Failure

Patients who have experienced treatment failure with a sofosbuvir-based regimen should be retreated with 12 weeks of sofosbuvir/velpatasvir/voxilaprevir. However, in patients with genotype 3 and cirrhosis, addition of ribavirin for 12 weeks is recommended. Sixteen weeks of glecaprevir/pibrentasvir is an alternative regimen [83]. Failure with multiple DAAs is likely due to RASs (resistance-associated substitutions) and warrants resistance analysis.

## 18. HCV Treatment in Special Populations

It is important to note that certain patient populations pose particular challenges in treating HCV. HIV/HCV coinfected patients have higher morbidity and mortality compared to monoinfected HCV patients. Additionally, HIV infection is independently associated with advanced liver fibrosis [84,85]. Many studies show similar efficacy and side effects when treated with DAA in HIV/HCV coinfected patients compared to monoinfected HCV patients; however, drug interactions need to be closely monitored [86,87,88,89].

All pregnant women should be screened for HCV during each pregnancy in accordance with the USPTF guidelines [90]. It is recommended that women of reproductive age with chronic HCV should be counselled and started on DAA therapy to prevent a risk of mother-to-child transmission. Studies suggest that pregnancy does not negatively affect chronic HCV infection [91,92]. Currently, there are no large clinical trials evaluating the safety of DAA (HCV treatment guidelines) during pregnancy, and starting DAA during pregnancy can be based on an individual discussion between patient and physician [93].

Chronic HCV is associated with an increased risk of proteinuria and increased incidence of CKD [94,95]. However, no dose adjustment is needed to DAA therapy for patients with CKD when treated with appropriate regimens.

Patients with decompensated cirrhosis, moderate or severe hepatic impairment, or Child–Turcotte–Pugh (CTP) class B or class C should ideally be treated by a specialist trained in treating advanced liver disease or at a liver transplant center. Studies suggest that patients with a Model for End-Stage Liver Disease (MELD) > 20 or severe portal hypertension may not improve with DAA therapy and may benefit from liver transplantation [96,97]. One of the major side effects of ribavirin is anemia, and it is more common in patients with decompensated cirrhosis. These patients should be started at a lower dose of ribavirin when initiating therapy [78].

## 19. Conclusions

PCPs play an important role in HCV diagnosis and management as they are often the first source of care for many patients. To minimize the transmission and complications of HCV, it is critical that PCPs identify individuals at risk, conduct appropriate screening, and ensure a prompt diagnosis of HCV. Timely treatment of HCV patients can reduce the risk of complications such as cirrhosis and HCC. Once a patient starts treatment for HCV, PCPs should follow up with them throughout the treatment to ensure that the patients reach SVR12. After treatment completion, patients should be followed up to monitor for any signs of hepatic and extrahepatic complications, in addition to any signs of HCV reinfection. With a proper understanding of HCV screening, diagnosis, treatment, and follow-up guidelines, PCPs can play a crucial role in HCV elimination.

## Figures and Tables

**Table 1:** Table of HCV treatment regimens by genotype.

Genotype	Condition	HCV Treatment Regimens
Genotype 1a	Well Compensated or No Cirrhosis	- Glecaprevir/pibrentasvir (Mavyret) 300/120 mg once daily for 8 weeks - Ledipasvir/sofosbuvir (Harvoni) 90/400 mg once daily for 12 weeks - Sofosbuvir/velpatasvir (Epclusa) 400/100 mg once daily for 12 weeks - Ledipasvir/Sofosbuvir 90/400 for 8 weeks for HIV-uninfected and HCV RNA < 6 million IU/ml - Elbasvir/grazoprevir (Zepatier) 50/100 mg once daily for 12 weeks (alternate)
Genotype 1b	Well Compensated or No Cirrhosis	- Glecaprevir/pibrentasvir (Mavyret) 300/120 mg once daily for 8 weeks - Ledipasvir/sofosbuvir (Harvoni) 90/400 mg once daily for 12 weeks - Sofosbuvir/velpatasvir (Epclusa) 400/100 mg once daily for 12 weeks - Ledipasvir/Sofosbuvir 90/400 for 8 weeks for HIV-uninfected and HCV RNA < 6 million IU/ml - Elbasvir/grazoprevir (Zepatier) 50/100 mg once daily for 12 weeks
Genotype 1	Decompensated Liver Disease	- Paritaprevir 150 mg + ritonavir 100 mg + ombitasvir 25 mg + dasabuvir 250 mg twice daily + ribavirin for 12 weeks - Sofosbuvir 400 mg/ledipasvir 90 mg + low-dose ribavirin (600 mg; increase as tolerated) for 12 weeks - Sofosbuvir 400 mg/velpatasvir 100 mg + weight-based ribavirin for 12 weeks
Genotype 2	Well Compensated or No Cirrhosis	- Glecaprevir/pibrentasvir (Mavyret) 300/120 mg once daily for 8 weeks - Sofosbuvir/velpatasvir (Epclusa) 400/100 mg once daily for 12 weeks
	Decompensated Liver Disease	- Sofosbuvir 400 mg/velpatasvir 100 mg + weight-based ribavirin for 12 weeks
Genotype 3	Well Compensated or No Cirrhosis	- Glecaprevir/pibrentasvir (Mavyret) 300/120 mg once daily for 8 weeks - Sofosbuvir/velpatasvir (Epclusa) 400/100 mg once daily for 12 weeks - Sofosbuvir/daclatasvir (Sovaldi) 400/60 mg once daily +/− RBV (compensated cirrhosis) for 12 weeks
	Decompensated Liver Disease	- Sofosbuvir 400 mg/velpatasvir 100 mg + weight-based ribavirin for 12 weeks
Genotype 4	Well Compensated or No Cirrhosis	- Glecaprevir/pibrentasvir (Mavyret) 300/120 mg once daily for 8 weeks - Sofosbuvir/velpatasvir (Epclusa) 400/100 mg once daily for 12 weeks - Ledipasvir/sofosbuvir (Harvoni) 90/400 mg once daily for 12 weeks - Elbasvir/grazoprevir (Zepatier) 50/100 mg once daily for 12 weeks
	Decompensated Liver Disease	- Sofosbuvir 400 mg/ledipasvir 90 mg + low-dose ribavirin (600 mg; increase as tolerated) for 12 weeks - Sofosbuvir 400 mg/velpatasvir 100 mg + weight-based ribavirin for 12 weeks
Genotypes 5 and 6	Well Compensated or No Cirrhosis	- Glecaprevir/pibrentasvir (Mavyret) 300/120 mg once daily for 8 weeks - Sofosbuvir/velpatasvir (Epclusa) 400/100 mg once daily for 12 weeks - Ledipasvir/sofosbuvir (Harvoni) 90/400 mg once daily for 12 weeks
	Decompensated Liver Disease	- Sofosbuvir 400 mg/ledipasvir 90 mg + low-dose ribavirin (600 mg; increase as tolerated) for 12 weeks - Sofosbuvir 400 mg/velpatasvir 100 mg + weight-based ribavirin for 12 weeks
Genotypes 1–6	Renal Failure (CrCl < 30 mL/min)	No dose adjustment in direct-acting antivirals is required when using recommended regimens
Genotypes 1–6	Decompensated Cirrhosis—Ribavirin Ineligible	- Sofosbuvir/velpatasvir (Epclusa) 400/100 mg once daily for 24 weeks
Genotype 1, 4, 5, or 6 Only	Decompensated Cirrhosis—Ribavirin Ineligible	- Ledipasvir/sofosbuvir (Harvoni) 90/400 mg once daily for 24 weeks
Cautions for Concurrent PPI Use		- PPIs may lower ledipasvir, so administer 4 hours before - PPI may increase concentrations of ombitasvir and dasabuvir—8 weeks for patients without HIV with HCV RNA level < 6 million IU/mL

## References

[1] Tsoulfas G., Goulis I., Giakoustidis D., Akriviadis E., Agorastou P., Imvrios G., Papanikolaou V. (2009). Hepatitis C and liver transplantation. Hippokratia.

[2] Cunningham E.B., Applegate T.L., Lloyd A.R., Dore G.J., Grebely J. (2015). Mixed HCV infection and reinfection in people who inject drugs—impact on therapy. Nat. Rev. Gastroenterol. Hepatol..

[3] Rosenberg E.S., Rosenthal E.M., Hall E.W., Barker L., Hofmeister M.G., Sullivan P.S., Dietz P., Mermin J., Ryerson A.B. (2018). Prevalence of Hepatitis C Virus Infection in US States and the District of Columbia, 2013 to 2016. JAMA Netw. Open.

[4] Lewis K.C., Barker L.K., Jiles R.B., Gupta N. (2023). Estimated Prevalence and Awareness of Hepatitis C Virus Infection Among US Adults: National Health and Nutrition Examination Survey, January 2017–March 2020. Clin. Infect. Dis..

[5] Cox A.L., El-Sayed M.H., Kao J.H., Lazarus J.V., Lemoine M., Lok A.S., Zoulim F. (2020). Progress towards elimination goals for viral hepatitis. Nat. Rev. Gastroenterol. Hepatol..

[6] World Health Organization Guidelines for the Care and Treatment of Persons Diagnosed with Chronic Hepatitis C Virus Infection. https://iris.who.int/bitstream/handle/10665/273174/9789241550345-eng.pdf?sequence=1.

[7] Saito T., Ueno Y. (2013). Transmission of hepatitis C virus: Self-limiting hepatitis or chronic hepatitis?. World J. Gastroenterol..

[8] Brunner N., Bruggmann P. (2021). Trends of the Global Hepatitis C Disease Burden: Strategies to Achieve Elimination. J. Prev. Med. Public Health.

[9] Bartenschlager R., Frese M., Pietschmann T. (2004). Novel insights into hepatitis C virus replication and persistence. Adv. Virus Res..

[10] Lindenbach B.D., Rice C.M. (2005). Unravelling hepatitis C virus replication from genome to function. Nature.

[11] Kiser J.J., Flexner C. (2013). Direct-acting antiviral agents for hepatitis C virus infection. Annu. Rev. Pharmacol. Toxicol..

[12] Martinello M., Naggie S., Rockstroh J.K., Matthews G.V. (2023). Direct-Acting Antiviral Therapy for Treatment of Acute and Recent Hepatitis C Virus Infection: A Narrative Review. Clin. Infect. Dis..

[13] Aghemo A., Piroth L., Bhagani S. (2018). What do clinicians need to watch for with direct-acting antiviral therapy?. J. Int. AIDS Soc..

[14] Schwarz T., Horváth I., Fenz L., Schmutterer I., Rosian-Schikuta I., Mårdh O. (2022). Interventions to increase linkage to care and adherence to treatment for hepatitis C among people who inject drugs: A systematic review and practical considerations from an expert panel consultation. Int. J. Drug Policy.

[15] Spaulding A.C., Weinbaum C.M., Lau D.T., Sterling R., Seeff L.B., Margolis H.S., Hoofnagle J.H. (2006). A framework for management of hepatitis C in prisons. Ann. Intern. Med..

[16] Facente S.N., Burk K., Eagen K., Mara E.S., Smith A.A., Lynch C.S. (2018). New Treatments Have Changed the Game: Hepatitis C Treatment in Primary Care. Infect. Dis. Clin. N. Am..

[17] Rattay T., Dumont I.P., Heinzow H.S., Hutton D.W. (2017). Cost-Effectiveness of Access Expansion to Treatment of Hepatitis C Virus Infection Through Primary Care Providers. Gastroenterology.

[18] Simoncini G.M., Koren D.E. (2019). Hepatitis C Update and Expanding the Role of Primary Care. J. Am. Board Fam. Med..

[19] Lohia P., Kapur S., Crane L. (2022). A Comprehensive Hepatitis C Treatment Program—An Observational Study of Collaboration Between Infectious Disease Specialists and General Internal Medicine Provider Serving a Majority Black Population. Infect. Dis. Clin. Pract..

[20] Guss D., Sherigar J., Rosen P., Mohanty S.R. (2018). Diagnosis and Management of Hepatitis C Infection in Primary Care Settings. J. Gen. Intern. Med..

[21] Oru E., Trickey A., Shirali R., Kanters S., Easterbrook P. (2021). Decentralisation, integration, and task-shifting in hepatitis C virus infection testing and treatment: A global systematic review and meta-analysis. Lancet Glob. Health.

[22] Kattakuzhy S., Gross C., Emmanuel B., Teferi G., Jenkins V., Silk R., Akoth E., Thomas A., Ahmed C., Espinosa M. (2017). Expansion of Treatment for Hepatitis C Virus Infection by Task Shifting to Community-Based Nonspecialist Providers: A Nonrandomized Clinical Trial. Ann. Intern. Med..

[23] Majagi A., Almujarkesh M.K., Carter K.S., Picardal A., Kapur S., Levine D.L., Lohia P. (2024). June. A.B. Hepatitis c: Everything a primary care physician needs to know about hepatitis c from diagnosis to long term follow up. J. Gen. Intern. Med..

[24] World Health Organization Hepatitis C. https://www.who.int/news-room/fact-sheets/detail/hepatitis-c.

[25] Petruzziello A., Marigliano S., Loquercio G., Cozzolino A., Cacciapuoti C. (2016). Global epidemiology of hepatitis C virus infection: An up-date of the distribution and circulation of hepatitis C virus genotypes. World J. Gastroenterol..

[26] Centers for Disease Control and Prevention (2022). Reduce Reported Rate of Hepatitis C-Related Deaths by 20% or More. https://www.cdc.gov/hepatitis/policy/npr/2022/reduce-reported-hepatitis-c-deaths.htm.

[27] Hall E.W., Schillie S., Vaughan A.S., Jones J., Bradley H., Lopman B., Rosenberg E.S., Sullivan P.S. (2021). County-Level Variation in Hepatitis C Virus Mortality and Trends in the United States, 2005–2017. Hepatology.

[28] Grebely J., Prins M., Hellard M., Cox A.L., Osburn W.O., Lauer G., Page K., Lloyd A.R., Dore G.J. (2012). Hepatitis C virus clearance, reinfection, and persistence, with insights from studies of injecting drug users: Towards a vaccine. Lancet. Infect. Dis..

[29] Chandra N., Joshi N., Raju Y.S., Kumar A., Teja V.D. (2013). Hepatitis B and/or C co-infection in HIV infected patients: A study in a tertiary care centre from South India. Indian J. Med. Res..

[30] Schillie S., Wester C., Osborne M., Wesolowski L., Ryerson A.B. (2020). CDC Recommendations for Hepatitis C Screening Among Adults—United States, 2020. MMWR Recomm. Rep..

[31] Cox A.L., Netski D.M., Mosbruger T., Sherman S.G., Strathdee S., Ompad D., Vlahov D., Chien D., Shyamala V., Ray S.C. (2005). Prospective evaluation of community-acquired acute-phase hepatitis C virus infection. Clin. Infect. Dis..

[32] Poynard T., Bedossa P., Opolon P. (1997). Natural history of liver fibrosis progression in patients with chronic hepatitis C. The OBSVIRC, METAVIR, CLINIVIR, and DOSVIRC groups. Lancet.

[33] Thomson E.C., Nastouli E., Main J., Karayiannis P., Eliahoo J., Muir D., McClure M.O. (2009). Delayed anti-HCV antibody response in HIV-positive men acutely infected with HCV. AIDS.

[34] Vanhommerig J.W., Thomas X.V., van der Meer J.T.M., Geskus R.B., Bruisten S.M., Molenkamp R., Prins M., Schinkel J. (2014). Hepatitis C virus (HCV) antibody dynamics following acute HCV infection and reinfection among HIV-infected men who have sex with men. Clin. Infect. Dis..

[35] Centers for Disease Control and Prevention (2023). Clinical Screening and Diagnosis for Hepatitis C. https://www.cdc.gov/hepatitis-c/hcp/diagnosis-testing/index.html.

[36] Gupta E., Bajpai M., Choudhary A. (2014). Hepatitis C virus: Screening, diagnosis, and interpretation of laboratory assays. Asian J. Transfus. Sci..

[37] Lingala S., Ghany M.G. (2015). Natural History of Hepatitis C. Gastroenterol. Clin. N. Am..

[38] Thein H.H., Yi Q., Dore G.J., Krahn M.D. (2008). Estimation of stage-specific fibrosis progression rates in chronic hepatitis C virus infection: A meta-analysis and meta-regression. Hepatology.

[39] Sarin S.K., Kumar M. (2012). Natural history of HCV infection. Hepatol. Int..

[40] Everhart J.E., Wright E.C., Goodman Z.D., Dienstag J.L., Hoefs J.C., Kleiner D.E., Ghany M.G., Mills A.S., Nash S.R., Govindarajan S. (2010). Prognostic value of Ishak fibrosis stage: Findings from the hepatitis C antiviral long-term treatment against cirrhosis trial. Hepatology.

[41] Mari P.C., Gulati R., Fragassi P. (2021). Adolescent Hepatitis C: Prevalence, Impact, and Management Challenges. Adolesc. Health Med. Ther..

[42] Pradat P., Voirin N., Tillmann H.L., Chevallier M., Trépo C. (2007). Progression to cirrhosis in hepatitis C patients: An age-dependent process. Liver Int..

[43] Mehta S.H., Brancati F.L., Sulkowski M.S., Strathdee S.A., Szklo M., Thomas D.L. (2000). Prevalence of type 2 diabetes mellitus among persons with hepatitis C virus infection in the United States. Ann. Intern. Med..

[44] Agnello V., Chung R.T., Kaplan L.M. (1992). A role for hepatitis C virus infection in type II cryoglobulinemia. N. Engl. J. Med..

[45] Gill K., Ghazinian H., Manch R., Gish R. (2016). Hepatitis C virus as a systemic disease: Reaching beyond the liver. Hepatol. Int..

[46] Phelan M., Cook C. (2014). A treatment revolution for those who can afford it? Hepatitis C treatment: New medications, profits and patients. BMC Infect. Dis..

[47] Ippolito G., Capobianchi M.R., Lanini S., Antonelli G. (2015). Is hepatitis C virus eradication around the corner only 25 years after its discovery?. Int. J. Antimicrob. Agents.

[48] Brzdęk M., Zarębska-Michaluk D., Invernizzi F., Cilla M., Dobrowolska K., Flisiak R. (2023). Decade of optimizing therapy with direct-acting antiviral drugs and the changing profile of patients with chronic hepatitis C. World J. Gastroenterol..

[49] Kish T., Aziz A., Sorio M. (2017). Hepatitis C in a New Era: A Review of Current Therapies. Pharm. Ther..

[50] Lee S.W., Lee T.Y., Yang S.S., Peng Y.C., Yeh H.Z., Chang C.S. (2018). Prevalence of Hepatitis B Reactivation Among Chinese Individuals with Chronic Hepatitis C Treated with Pan-Oral Direct-Acting Antivirals. Gastroenterol. Res..

[51] Mavilia M.G., Wu G.Y. (2018). HBV-HCV Coinfection: Viral Interactions, Management, and Viral Reactivation. J. Clin. Transl. Hepatol..

[52] Mücke M.M., Backus L.I., Mücke V.T., Coppola N., Preda C.M., Yeh M.-L., Tang L.S.Y., Belperio P.S., Wilson E.M., Yu M.-L. (2018). Hepatitis B virus reactivation during direct-acting antiviral therapy for hepatitis C: A systematic review and meta-analysis. Lancet Gastroenterol. Hepatol..

[53] University of Liverpool Interactions with HCV DAAs and Ribavirin. www.hep-druginteractions.org/prescribing_resources/hep-summaries-hcv.

[54] Norton B.L., Fleming J., Bachhuber M.A., Steinman M., DeLuca J., Cunningham C.O., Johnson N., Laraque F., Litwin A.H. (2017). High HCV cure rates for people who use drugs treated with direct acting antiviral therapy at an urban primary care clinic. Int. J. Drug Policy.

[55] Hui V.W.-K., Au C.L., Lam A.S.M., Yip T.C.-F., Tse Y.-K., Lai J.C.-T., Chan H.L.-Y., Wong V.W.-S., Wong G.L.-H. (2022). Drug-drug interactions between direct-acting antivirals and co-medications: A territory-wide cohort study. Hepatol. Int..

[56] Hayes K.N., Burkard T., Weiler S., Tadrous M., Burden A.M. (2021). Global adverse events reported for direct-acting antiviral therapies for the treatment of hepatitis C: An analysis of the World Health Organization VigiBase. Eur. J. Gastroenterol. Hepatol..

[57] Manns M.P., Pockros P.J., Norkrans G., Smith C.I., Morgan T.R., Häussinger D., Shiffman M.L., Hadziyannis S.J., Schmidt W.N., Jacobson I.M. (2013). Long-term clearance of hepatitis C virus following interferon α-2b or peginterferon α-2b, alone or in combination with ribavirin. J. Viral Hepat..

[58] Swain M.G., Lai M.-Y., Shiffman M.L., Cooksley W.G.E., Zeuzem S., Dieterich D.T., Abergel A., Pessôa M.G., Lin A., Tietz A. (2010). A sustained virologic response is durable in patients with chronic hepatitis C treated with peginterferon alfa-2a and ribavirin. Gastroenterology.

[59] Marinho R.T., Vitor S., Velosa J. (2014). Benefits of curing hepatitis C infection. J. Gastrointestin. Liver Dis..

[60] Fabrizi F., Dixit V., Messa P. (2013). Antiviral therapy of symptomatic HCV-associated mixed cryoglobulinemia: Meta-analysis of clinical studies. J. Med. Virol..

[61] Maddison A.R., Fisher J., Johnston G. (2011). Preventive medication use among persons with limited life expectancy. Prog. Palliat. Care.

[62] Holmes H.M., Hayley D.C., Alexander G.C., Sachs G.A. (2006). Reconsidering medication appropriateness for patients late in life. Arch. Intern. Med..

[63] Razavi H., Elkhoury A.C., Elbasha E., Estes C., Pasini K., Poynard T., Kumar R. (2013). Chronic hepatitis C virus (HCV) disease burden and cost in the United States. Hepatology.

[64] U.S. Senate Committee on Finance (2015). The Price of Sovaldi and Its Impact on the US Health Care System. https://www.finance.senate.gov/download/the-price-of-sovaldi-and-its-impact-on-the-us-health-care-system-full-report.

[65] Silseth S., Shaw H. (2021). Analysis of Prescription Drugs for the Treatment of Hepatitis C in the United States. https://edge.sitecorecloud.io/millimaninc5660-milliman6442-prod27d5-0001/media/Milliman/PDFs/2021-Articles/6-11-21-Analysis-prescription-drugs-treatment-hepatitis-C-US.pdf.

[66] He T., Lopez-Olivo M.A., Hur C., Chhatwal J. (2017). Systematic review: Cost-effectiveness of direct-acting antivirals for treatment of hepatitis C genotypes 2-6. Aliment. Pharmacol. Ther..

[67] Szabó E., Lotz G., Páska C., Kiss A., Schaff Z. (2003). Viral hepatitis: New data on hepatitis C infection. Pathol. Oncol. Res..

[68] Moreno R., Berenguer M. (2005). Liver transplantation in patients with chronic viral hepatitis. Rev. Gastroenterol. Mex..

[69] van der Hilst C.S., Ijtsma A.J., Slooff M.J., Tenvergert E.M. (2009). Cost of liver transplantation: A systematic review and meta-analysis comparing the United States with other OECD countries. Med. Care Res. Rev..

[70] Leidner A.J., Chesson H.W., Xu F., Ward J.W., Spradling P.R., Holmberg S.D. (2015). Cost-effectiveness of hepatitis C treatment for patients in early stages of liver disease. Hepatology.

[71] Artenie A.A., Bruneau J., Lévesque A., Wansuanganyi J.M. (2014). Role of primary care providers in hepatitis C prevention and care: One step away from evidence-based practice. Can. Fam. Physician.

[72] Ghany M.G., Morgan T.R., Panel A.-I. (2020). HCG. Hepatitis C Guidance 2019 Update: American Association for the Study of Liver Diseases-Infectious Diseases Society of America Recommendations for Testing, Managing, and Treating Hepatitis C Virus Infection. Hepatology.

[73] Strassl R., Rutter K., Stättermayer A.F., Beinhardt S., Kammer M., Hofer H., Ferenci P., Popow-Kraupp T. (2015). Real-Time PCR Assays for the Quantification of HCV RNA: Concordance, Discrepancies and Implications for Response Guided Therapy. PLoS ONE.

[74] Stauber R.E., Lackner C. (2007). Noninvasive diagnosis of hepatic fibrosis in chronic hepatitis C. World J. Gastroenterol..

[75] Nguyen D.L., Hu K.Q. (2014). Clinical Monitoring of Chronic Hepatitis C Based on its Natural History and Therapy. N. Am. J. Med. Sci..

[76] U.S. Food and Drug Administration (2015). FDA Drug Safety Communication: FDA Warns of Serious Slowing of the Heart Rate When Antiarrhythmic Drug Amiodarone Is Used with Hepatitis C Treatments Containing Sofosbuvir (Harvoni) or Sovaldi in Combination with Another Direct Acting Antiviral Drug. https://www.fda.gov/drugs/drug-safety-and-availability/fda-drug-safety-communication-fda-warns-serious-slowing-heart-rate-when-antiarrhythmic-drug.

[77] Morisco F., Granata R., Stroffolini T., Guarino M., Donnarumma L., Gaeta L., Loperto I., Gentile I., Auriemma F., Caporaso N. (2013). Sustained virological response: A milestone in the treatment of chronic hepatitis C. World J. Gastroenterol..

[78] AASLD, IDSA (2023). Monitoring Patients Who Are Starting HCV Treatment, Are on Treatment, or Have Completed Therapy. https://www.hcvguidelines.org/evaluate/monitoring.

[79] Sarrazin C., Isakov V., Svarovskaia E.S., Hedskog C., Martin R., Chodavarapu K., Brainard D.M., Miller M.D., Mo H., Molina J.-M. (2017). Late Relapse Versus Hepatitis C Virus Reinfection in Patients with Sustained Virologic Response After Sofosbuvir-Based Therapies. Clin. Infect. Dis..

[80] Simmons B., Saleem J., Hill A., Riley R.D., Cooke G.S. (2016). Risk of Late Relapse or Reinfection with Hepatitis C Virus After Achieving a Sustained Virological Response: A Systematic Review and Meta-analysis. Clin. Infect. Dis..

[81] Schneider M.D., Sarrazin C. (2016). Management of HCV-Associated Liver Cirrhosis. Visc. Med..

[82] Maness D.L., Riley E., Studebaker G. (2021). Hepatitis C: Diagnosis and Management. Am. Fam. Physician.

[83] AASLD, IDSA HCV Guidance: Recommendations for Testing, Managing, and Treating Hepatitis C. https://www.hcvguidelines.org/treatment-experienced/sof-and-elb-grz-failures.

[84] Re V.L., Kallan M.J., Tate J.P., Localio A.R., Lim J.K., Goetz M.B., Klein M.B., Rimland D., Rodriguez-Barradas M.C., Butt A.A. (2014). Hepatic decompensation in antiretroviral-treated patients co-infected with HIV and hepatitis C virus compared with hepatitis C virus-monoinfected patients: A cohort study. Ann. Intern. Med..

[85] Kirk G.D., Mehta S.H., Astemborski J., Galai N., Washington J., Higgins Y., Balagopal A., Thomas D.L. (2013). HIV, age, and the severity of hepatitis C virus-related liver disease: A cohort study. Ann. Intern. Med..

[86] Rockstroh J.K., Lacombe K., Viani R.M., Orkin C., Wyles D., Luetkemeyer A.F., Soto-Malave R., Flisiak R., Bhagani S., Sherman K.E. (2018). Efficacy and Safety of Glecaprevir/Pibrentasvir in Patients Coinfected with Hepatitis C Virus and Human Immunodeficiency Virus Type 1: The EXPEDITION-2 Study. Clin. Infect. Dis..

[87] Wyles D., Bräu N., Kottilil S., Daar E.S., Ruane P., Workowski K., Luetkemeyer A., Adeyemi O., Kim A.Y., Doehle B. (2017). Sofosbuvir and Velpatasvir for the Treatment of Hepatitis C Virus in Patients Coinfected with Human Immunodeficiency Virus Type 1: An Open-Label, Phase 3 Study. Clin. Infect. Dis..

[88] Naggie S., Cooper C., Saag M., Workowski K., Ruane P., Towner W.J., Marks K., Luetkemeyer A., Baden R.P., Sax P.E. (2015). Ledipasvir and Sofosbuvir for HCV in Patients Coinfected with HIV-1. N. Engl. J. Med..

[89] Bhattacharya D., Belperio P.S., Shahoumian T.A., Loomis T.P., Goetz M.B., Mole L.A., Backus L.I. (2017). Effectiveness of All-Oral Antiviral Regimens in 996 Human Immunodeficiency Virus/Hepatitis C Virus Genotype 1-Coinfected Patients Treated in Routine Practice. Clin. Infect. Dis..

[90] Owens D.K., Davidson K.W., Krist A.H., Barry M.J., Cabana M., Caughey A.B., Donahue K., Doubeni C.A., Epling J.W., Kubik M. (2020). Screening for Hepatitis C Virus Infection in Adolescents and Adults: US Preventive Services Task Force Recommendation Statement. JAMA.

[91] Conte D., Fraquelli M., Prati D., Colucci A., Minola E. (2000). Prevalence and clinical course of chronic hepatitis C virus (HCV) infection and rate of HCV vertical transmission in a cohort of 15,250 pregnant women. Hepatology.

[92] Gervais A., Bacq Y., Bernuau J., Martinot M., Auperin A., Boyer N., Kilani A., Erlinger S., Valla D., Marcellin P. (2000). Decrease in serum ALT and increase in serum HCV RNA during pregnancy in women with chronic hepatitis C. J. Hepatol..

[93] AASLD, IDSA (2023). HCV in Pregnancy. https://www.hcvguidelines.org/unique-populations/pregnancy.

[94] Rogal S.S., Yan P., Rimland D., Re V.L., Al-Rowais H., Fried L., Butt A.A. (2016). Incidence and Progression of Chronic Kidney Disease After Hepatitis C Seroconversion: Results from ERCHIVES. Dig. Dis. Sci..

[95] Fabrizi F., Verdesca S., Messa P., Martin P. (2015). Hepatitis C Virus Infection Increases the Risk of Developing Chronic Kidney Disease: A Systematic Review and Meta-Analysis. Dig. Dis. Sci..

[96] El-Sherif O., Jiang Z.G., Tapper E.B., Huang K.C., Zhong A., Osinusi A., Charlton M., Manns M., Afdhal N.H., Mukamal K. (2018). Baseline Factors Associated with Improvements in Decompensated Cirrhosis After Direct-Acting Antiviral Therapy for Hepatitis C Virus Infection. Gastroenterology.

[97] Terrault N.A., McCaughan G.W., Curry M.P., Gane E., Fagiuoli S., Fung J.Y.Y., Agarwal K., Lilly L., Strasser S.I., Brown K.A. (2017). International Liver Transplantation Society Consensus Statement on Hepatitis C Management in Liver Transplant Candidates. Transplantation.

